# Neuroimaging in Leprosy: A Case Series Exploring Domains Beyond Peripheral Nerves

**DOI:** 10.7759/cureus.81571

**Published:** 2025-04-01

**Authors:** Karunanithi Elangovan, Saurabh Kumar, Ravindra Kumar Garg, Anit Parihar, Kiranpreet Malhotra, Priyanka Verma, Deepika Rawat, Neeraj Kumar, Himanshu D Reddy, Swastika Suvirya

**Affiliations:** 1 Radiodiagnosis, King George's Medical University, Lucknow, IND; 2 Neurology, Era Medical College and Hospital, Lucknow, IND; 3 Pathology, Dr. Ram Manohar Lohia Institute of Medical Sciences, Lucknow, IND; 4 Dermatology, Venereology and Leprosy, King George's Medical University, Lucknow, IND; 5 Dermatology, Venereolgy and Leprosy, King George's Medical University, Lucknow, IND; 6 Neurology, King George's Medical University, Lucknow, IND; 7 Internal Medicine, King George's Medical University, Lucknow, IND

**Keywords:** brachial plexopathy, brain, cemri, cervical cord, leprosy, leprous ganglionitis, neuroimaging, signal intensity alteration

## Abstract

Background and objective

Leprosy, an infectious disease caused by *Mycobacterium leprae*, induces irreversible damage, necessitating early detection. The disease's neurotropism, which extends to the peripheral nerves, is well recognized. In recent years, there has been an acknowledgment of its expanding horizon beyond this traditional boundary.

This study aims to explore the involvement of atypical sites such as the central nervous system, brachial plexus, and spinal nerve root ganglion through dedicated neuroimaging via magnetic resonance imaging (MRI) across diverse spectra of leprosy.

Methods

In a retrospective case series conducted from July 2020 to July 2022 at a tertiary care hospital, leprosy patients with specific neurological signs were analyzed. Neurologists had recommended MRI for suspected central nervous system (CNS)/proximal nerve root involvement in these patients. Two expert radiologists assessed the MRI imaging results.

Results

Eleven such patients were identified, of which six with positive MRI findings were scrutinized. Six multi-drug therapy (MDT) naive male leprosy patients in the age range of 24-44 years were analyzed. MRI revealed signal intensity alterations (SIA) in the brain, cervical cord, and bilateral/unilateral brachial plexus/ganglion.

After a year of MDT, follow-up imaging revealed diverse responses: complete resolution of brain SIA (n=1), persistent declining hyperintensity in the cervical cord with declining (n=1), unchanged ganglionitis/plexitis (n=2), and no observable changes in cord and brachial plexus SIA (n=1).

Conclusion

This study reveals diverse neuroimaging patterns in leprosy patients. Neuroimaging may reveal abnormalities, particularly in patients with leprosy presenting with lower motor neuron-type facial palsy. A deeper understanding of these radiological findings could offer valuable insights into the pathophysiology of leprosy.

## Introduction

Leprosy, caused by *Mycobacterium leprae* or *lepromatosis*, has pernicious effects on peripheral nerves, skin, mucous membranes, and internal organs, resulting in permanent deformities and disabilities [1]. Traditionally, leprosy has been considered a disease of the peripheral nervous system; however, recent magnetic resonance imaging (MRI)-based studies have highlighted the involvement of the central nervous system (CNS) and nerve plexuses as well [1-8]. However, comprehensive characterization of these CNS lesions remains limited. While the evaluation of peripheral nerves often includes clinical assessment, ultrasonography, and biopsy, magnetic resonance imaging (MRI) plays a crucial role in assessing the involvement of the proximal nerve roots, plexus, brain, and spinal cord [2,9,10]. It provides detailed soft tissue contrast and facilitates a comprehensive assessment of the patient [11].

The objective of the study is to systematically investigate the neuroimaging characteristics in leprosy, including the central nervous system (CNS), proximal nerve roots, and brachial plexus, to better understand the diverse neuroimaging patterns associated with the disease.

## Materials and methods

This retrospective case series was conducted at a tertiary care hospital from July 2020 to July 2022. Approval for the same was obtained from the institutional ethical committee (125th ECM IIA/P16).

Clinical and imaging data were obtained from electronic and physical records. Patients meeting the inclusion criteria for this study were diagnosed with leprosy according to World Health Organization (WHO) criteria, classified via Ridley Jopling classification [12], and exhibited specific neurological findings, such as cranial nerve involvement or exaggerated deep tendon reflexes [2,9,11].

Exclusion criteria included diabetes, hypertension, alcoholism, malnutrition, toxin exposure, infections (hepatitis B, hepatitis C, human immunodeficiency virus), vasculitis, or malignancy. Written consent for the publication of medical data and clinical photographs was obtained from the treating physicians.

Out of 11 patients undergoing MRI, only six showed positive findings on the same. The recruited patients with findings on MRI were categorized based on the Ridley-Jopling classification as follows: three patients with borderline tuberculoid Hansen’s disease (BTHD), one with mid-borderline Hansen’s disease (BBHD), one with borderline lepromatous Hansen’s disease (BLHD), and one with lepromatous Hansen’s disease (LLHD).

MRI was performed on a General Electric (GE) SIGNA EXPLORER 1.5 Tesla MRI (Cincinnati, Ohio, USA) machine. Craniospinal MR imaging was conducted to evaluate the brain, spinal cord, brachial plexus, proximal nerve roots, and dorsal nerve root ganglia, utilizing a comprehensive range of sequences in axial, sagittal, and coronal planes, along with post-contrast imaging. For brain imaging, the MRI sequences acquired included axial T1-weighted (T1W), T2-weighted (T2W), and FLAIR sequences, as well as sagittal and coronal T2W, axial diffusion-weighted imaging (DWI), apparent diffusion coefficient (ADC), and susceptibility-weighted imaging (SWI). To assess the spinal cord, nerve roots, and ganglia, sagittal and axial T2W sequences, sagittal and axial T1W sequences, and coronal STIR sequences were utilized. Post-contrast imaging of the brain and spinal cord was performed using gadolinium-based contrast agents at a dosage of 0.1 mmol/kg, with post-contrast fat-suppressed T1W images obtained in axial, sagittal, and coronal planes of the brain and cervical spinal cord, including 3D BRAVO thin-section imaging specifically for the brain. There were no cases of pure neuritic leprosy in the final cohort of patients for analysis. For this study, long-segment hyperintense signals were defined when signals on MRI involved more than three cervical vertebral bodies. All patients had received multibacillary multi-drug therapy (MB-MDT) and oral steroids (prednisolone in a dose of 1 mg/kg and tapered by 10 mg every month for a total of 6-7 months in all six patients) as part of their disease treatment.

Details of follow-up in the form of clinical and radiological improvement were also analyzed. Five such MRIs were available for evaluation out of six patients.

## Results

**Figure 1:**
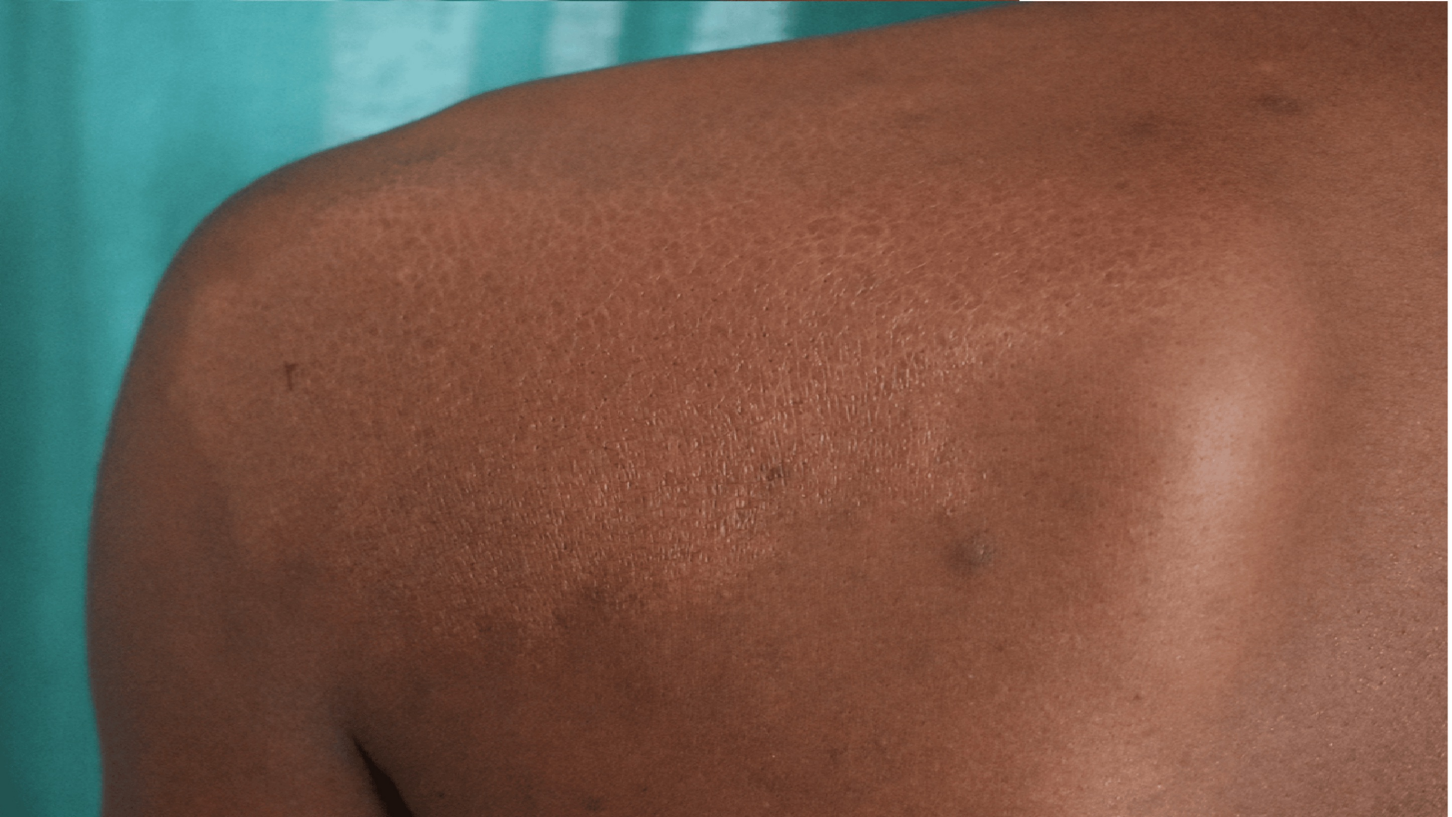
A large, well-defined, and hypo-pigmented to erythematous, shiny plaque measuring 15x10cm (case 3) A 29-year-old man with BTHD presenting with hypopigmented to erythematous, shiny plaque on the left scapula and left shoulder, left facial palsy, and Bell’s phenomenon.

**Figure 2:**
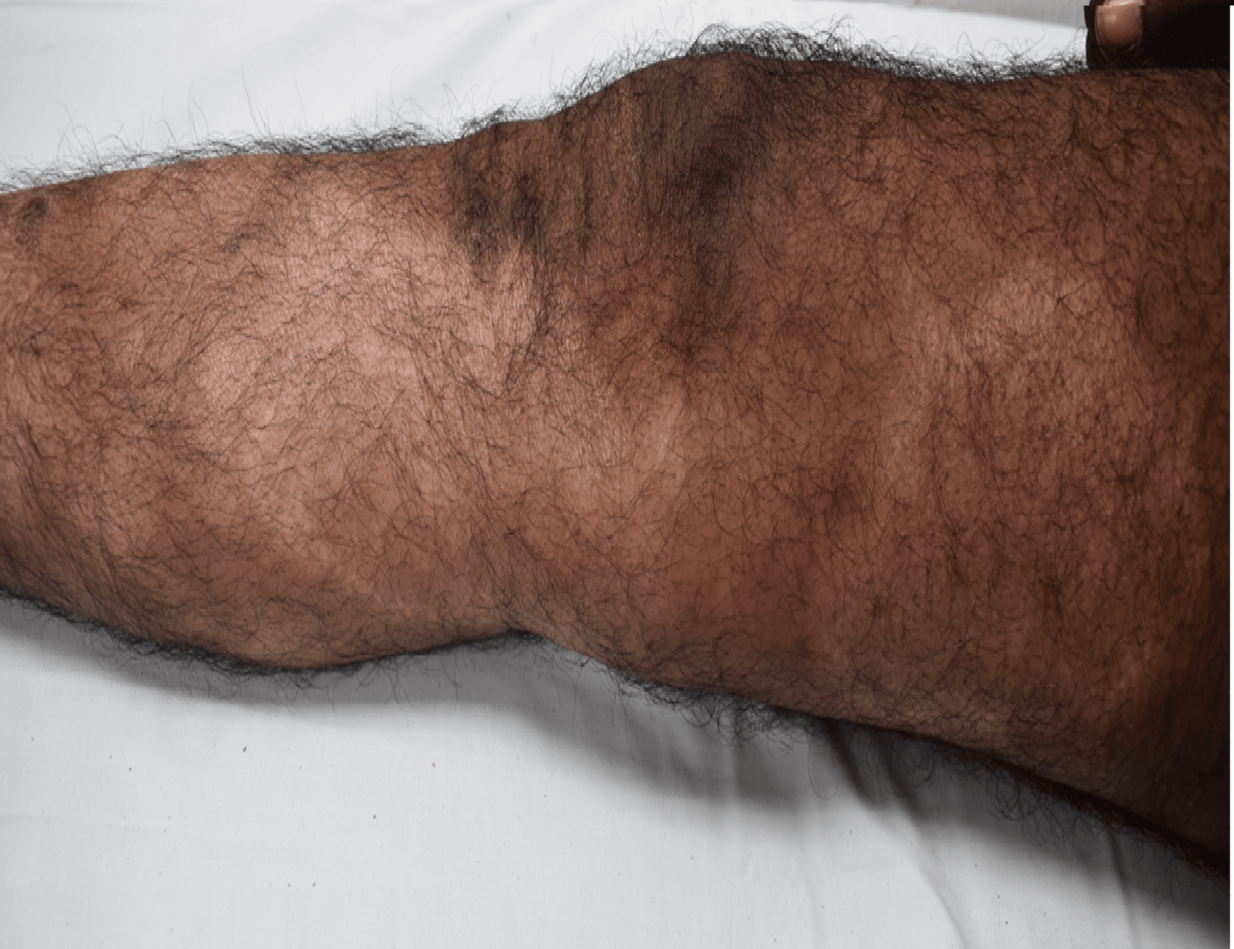
An annular patch with a hypopigmented border (7x10cm) on the right medial thigh and another hypopigmented patch extending from the medial aspect of the thigh to the lower leg (6x16cm) in the same patient (case 4) A 33-year-old male with BBHD presented with an annular patch with a hypopigmented border on the right thigh and right lower leg

**Figure 3:**
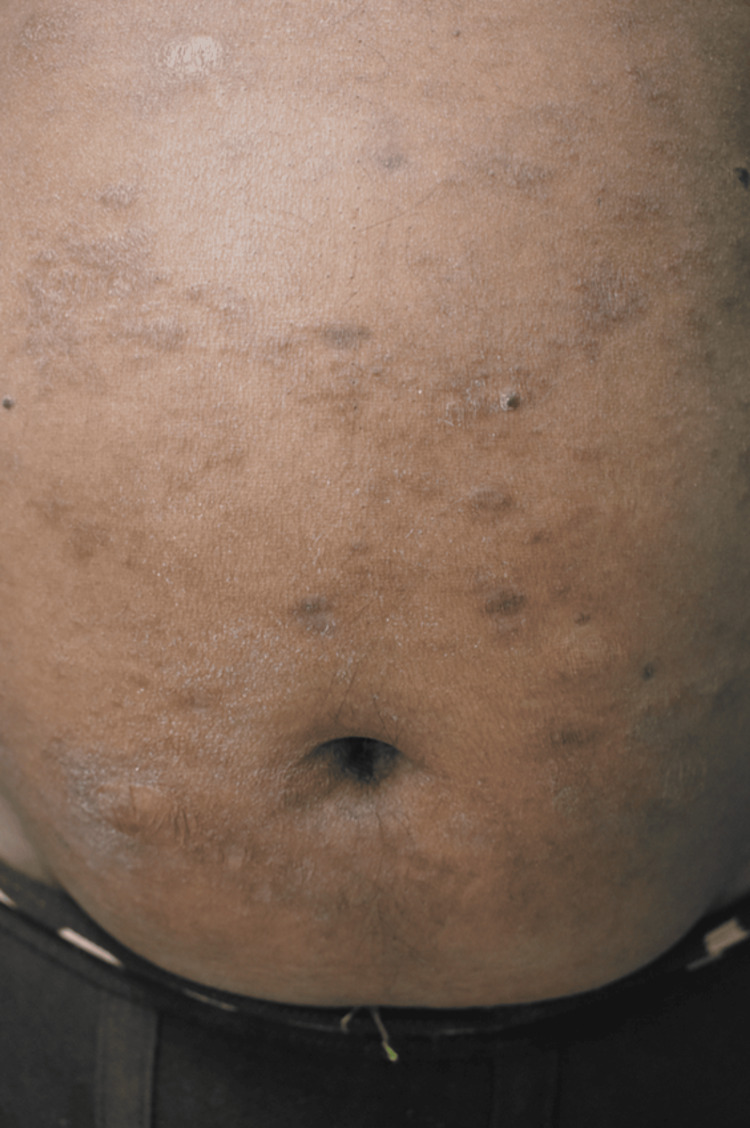
Multiple erythematous to violaceous, shiny, scaly nodules and plaques ranging in size from 1x1 cm to 1x2.5 cm were found over the abdomen (case 5) A 40-year-old male suffering from LLHD presented with left-sided facial palsy, infiltrated ear pinna, and multiple erythematous to violaceous, shiny, scaly nodules and plaques over the abdomen.

The study included six MDT-naïve male patients aged 24-44 years, with disease durations ranging from 1-5 years. Five patients exhibited hypopigmented and hypoesthetic skin lesions (Figures 1, 2), and one had nodular lesions (Figure 3).

**Figure 4:**
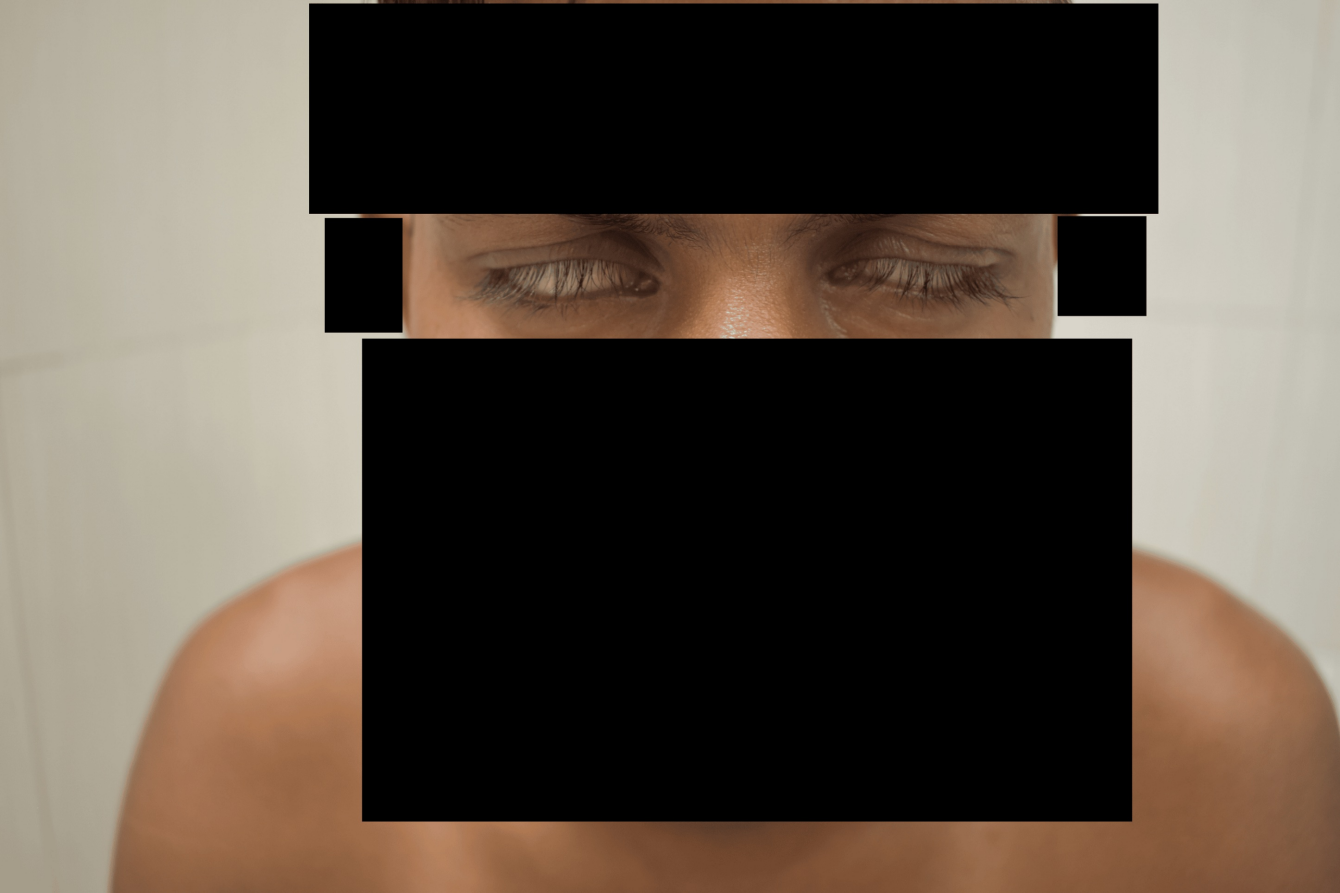
Bilateral Bell’s phenomenon (case 2) A 26-year-old man with BLHD presenting with bilateral Bell’s phenomenon and bilateral claw hands and toes.

**Figure 5:**
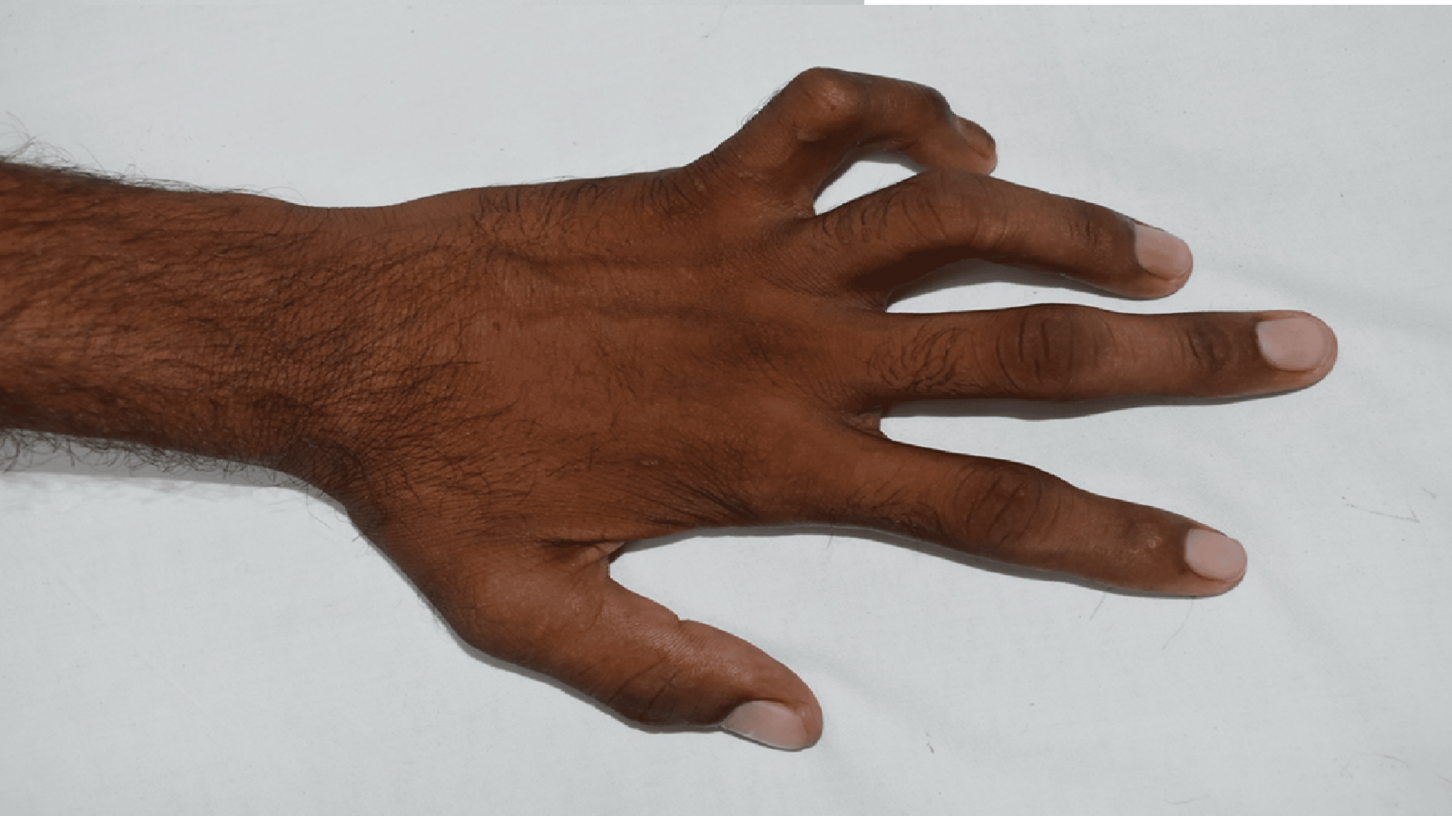
Partial clawing of left hand (case 1) A 24-year-old man with BTHD presented with a poorly defined hypopigmented patch on the left forearm and partial clawing in the left hand.

**Figure 6:**
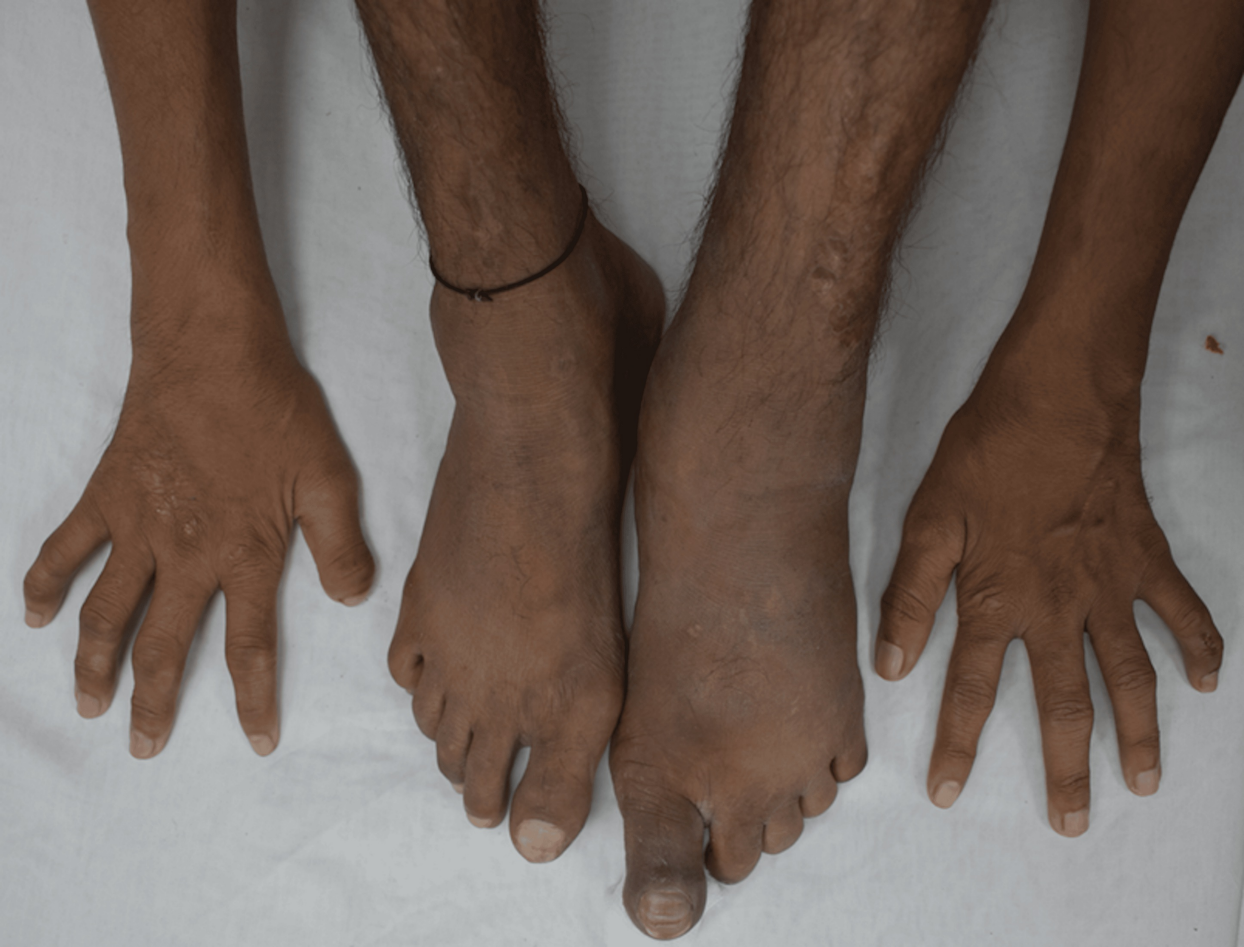
Bilateral claw hands and toes (case 2) A 26-year-old man with BLHD presenting with bilateral Bell’s phenomenon and bilateral claw hands and toes.

Motor deformities were present for a duration of 1-5 months, including facial nerve palsy (n=5) (Figure 4), unilateral claw hand (n=1) (Figure 5), and bilateral claw hands and toes (n=1) (Figure 6). Additional findings included exaggerated biceps and triceps reflexes on the left side (3+) (n=1) and loss of kinaesthetic perception (n=2).

Biochemical investigations were within normal limits, except for an increased erythrocyte sedimentation rate (ESR) (30 mm/hr) in one patient with a Type 1 reaction (T1R) and an elevated total leukocyte count (12,700/mm³) and ESR (50 mm/hr) in another with a Type 2 reaction (T2R). Slit skin smear positivity was observed in one patient. Patients were classified as Borderline Tuberculoid Hansen's Disease (BTHD) (n=3), Mid-Borderline Hansen's Disease (BBHD) (n=1), Borderline Lepromatous Hansen's Disease (BLHD) (n=1), and Lepromatous Hansen's Disease (LLHD) (n=1). A patient with BTHD had T1R for eight weeks, while another LLHD case had T2R for three weeks.

**Figure 7:**
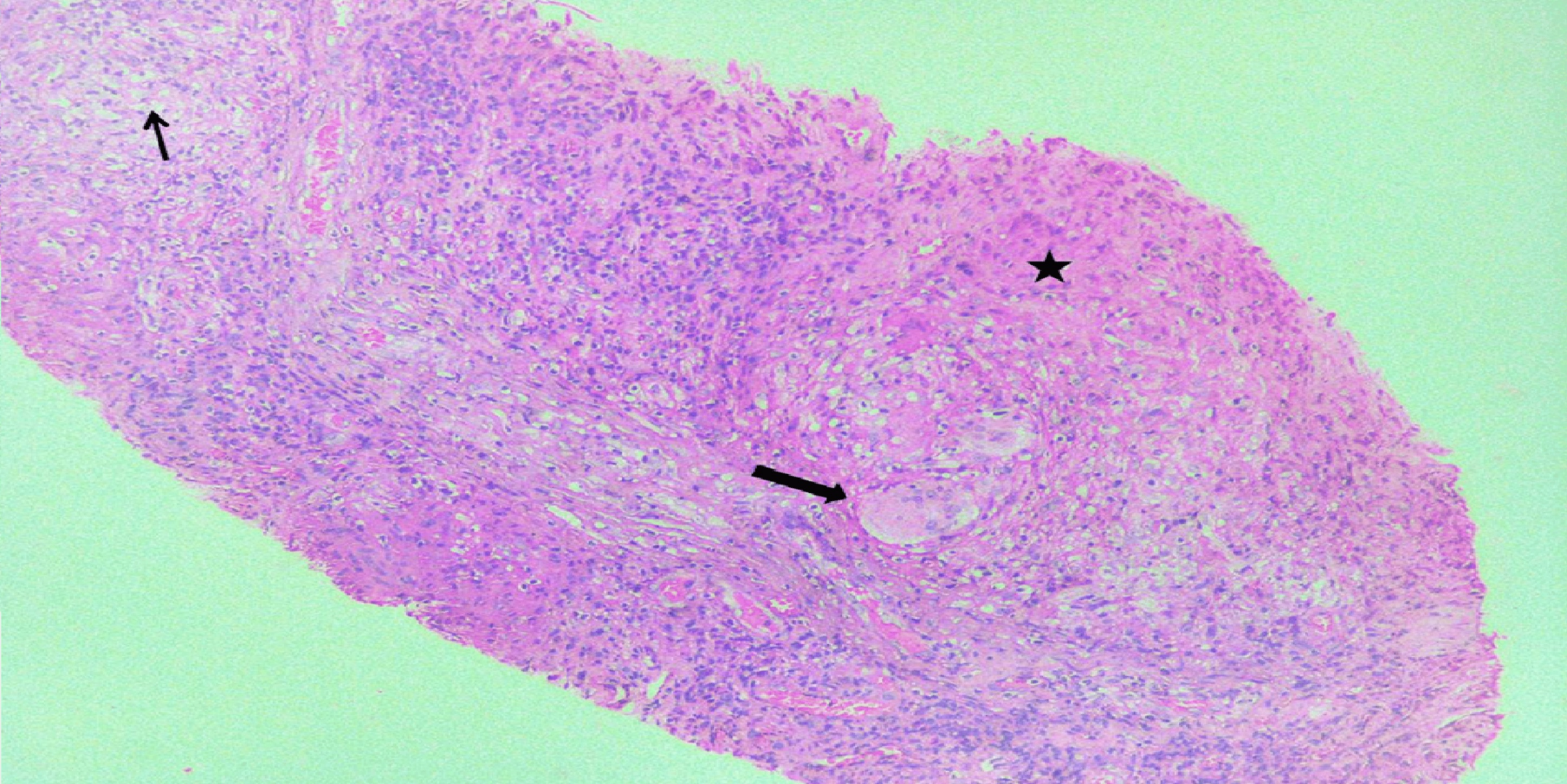
Section from the peripheral nerve showing an epithelioid granuloma (asterisk) with giant cells (bold arrow) and a few dispersed foamy macrophages (arrow). Hematoxylin & Eosin, x200 (case 4)

**Figure 8:**
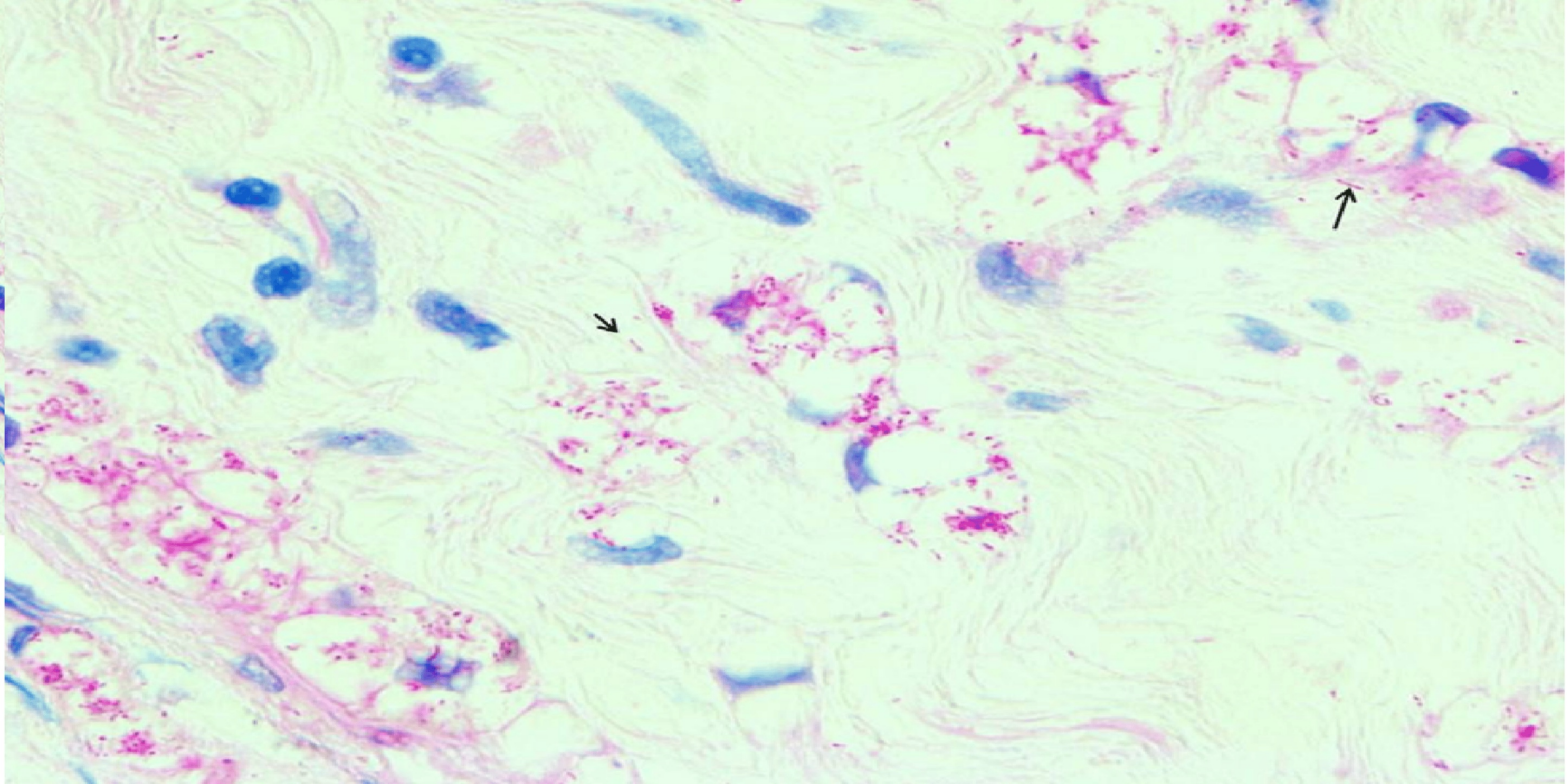
Section from peripheral nerve showing numerous lepra bacilli along with a globus (arrows), Wade Fite, x200 (case 5)

**Figure 9:**
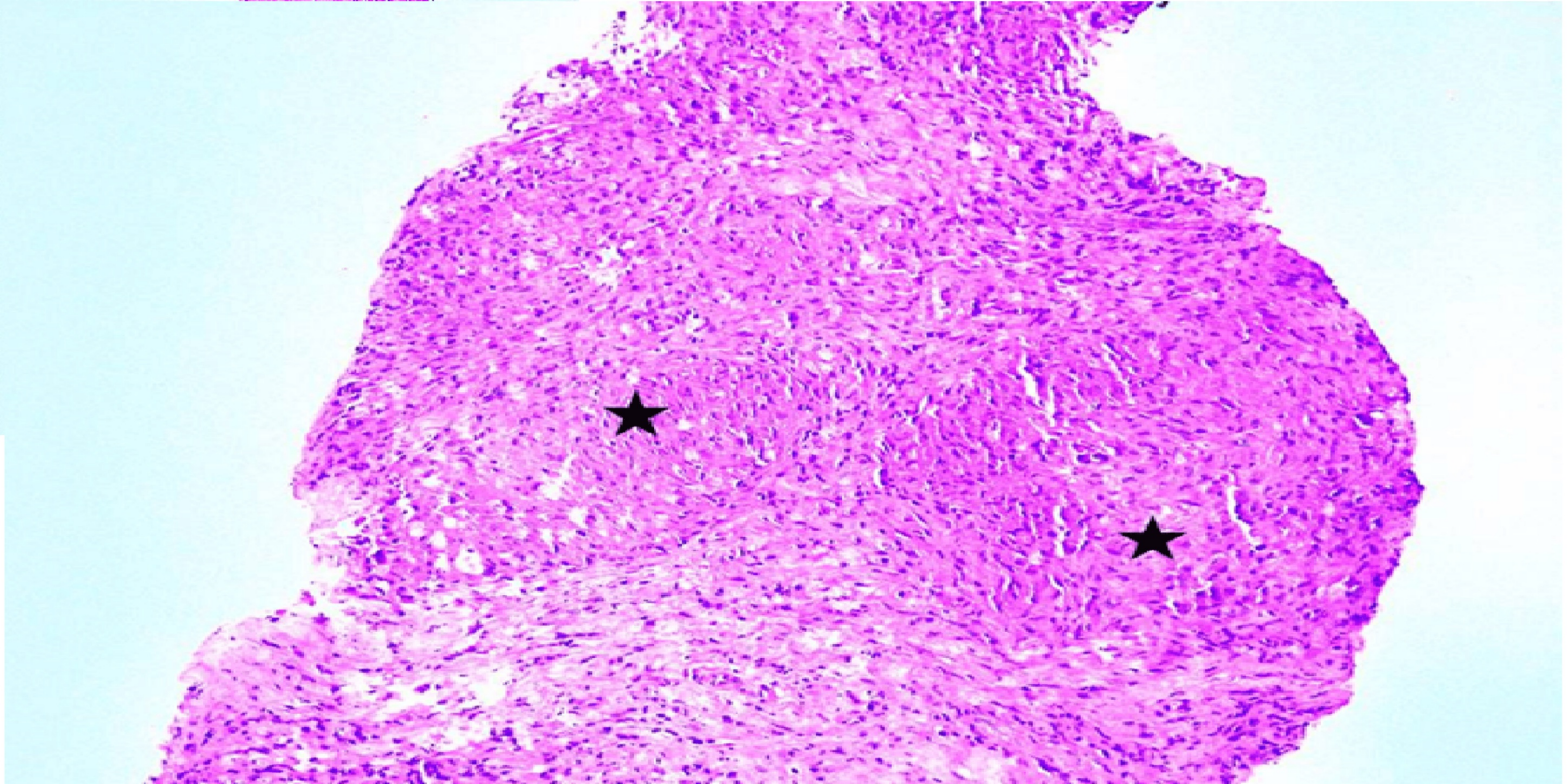
Section from peripheral nerve showing two epithelioid cell granulomas with interspersed lymphocytes (asterisk) Hematoxylin & Eosin, x200 (case 6)

The histological examination of skin and nerve samples showed consistent findings in the majority of patients, with three exceptions. Specifically, while the skin histopathology didn't indicate AFB positivity in the first scenario (BLHD), his nerve histopathology did reveal 0-10 bacilli/oil immersion field (OIF) positivity. In the case of BTHD and LLHD, the histopathological characteristics of T1R and T2R, respectively, were visible on the skin but not on the nerve (Figures 7-9).

MRI findings included signal intensity alterations in the brain (n=2) (Figures 10, 11), cervical cord (n=5) (Figures 12-15), bilateral brachial plexitis (n=2) (Figures 16, 17), and unilateral (Figure 18)/bilateral ganglionitis (n=3) (Figure 15). Post-contrast enhancement was present in five patients (Figures 19, 17).

**Figure 10:**
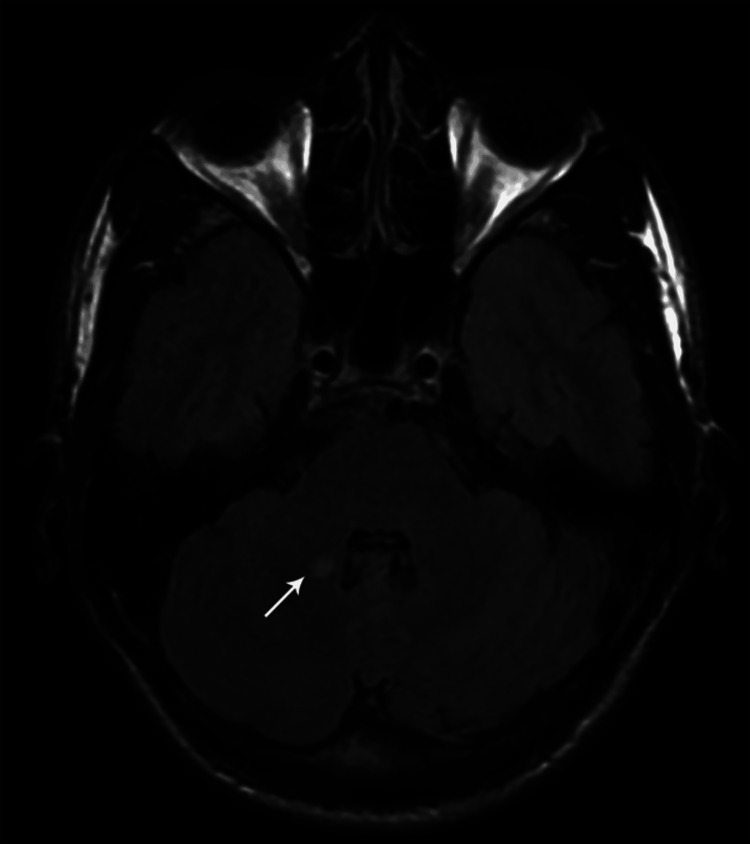
Axial T2 FLAIR MR brain image showing a focal hyperintense signal at the right middle cerebellar peduncle (case 1)

**Figure 11:**
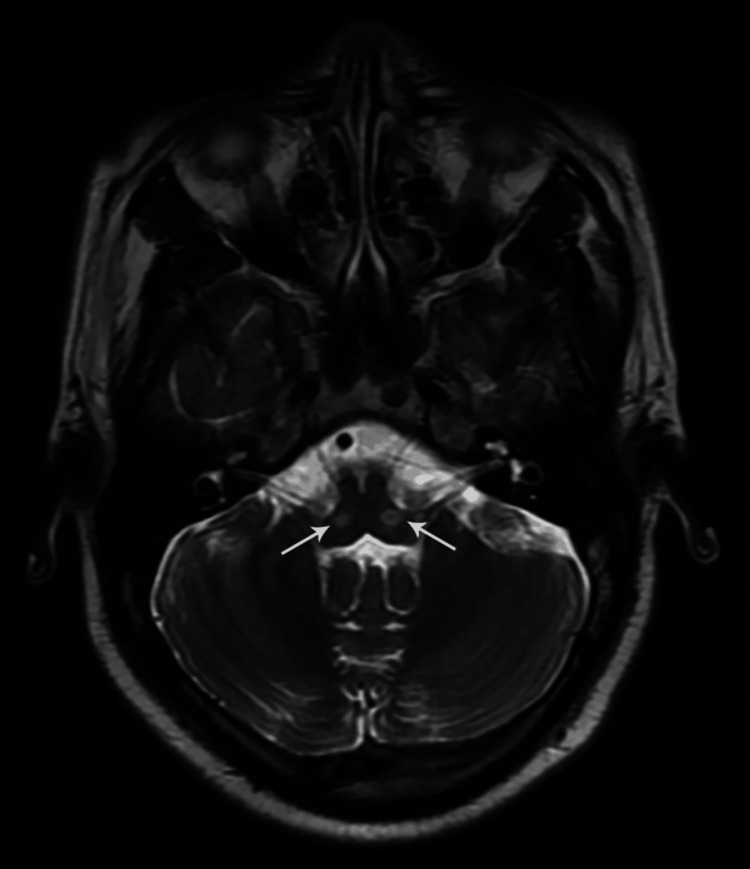
Axial T2W MR brain image showing bilaterally symmetrical hyperintense signals at the pontomedullary junction at the level of facial nerve nuclei (case 4)

**Figure 12:**
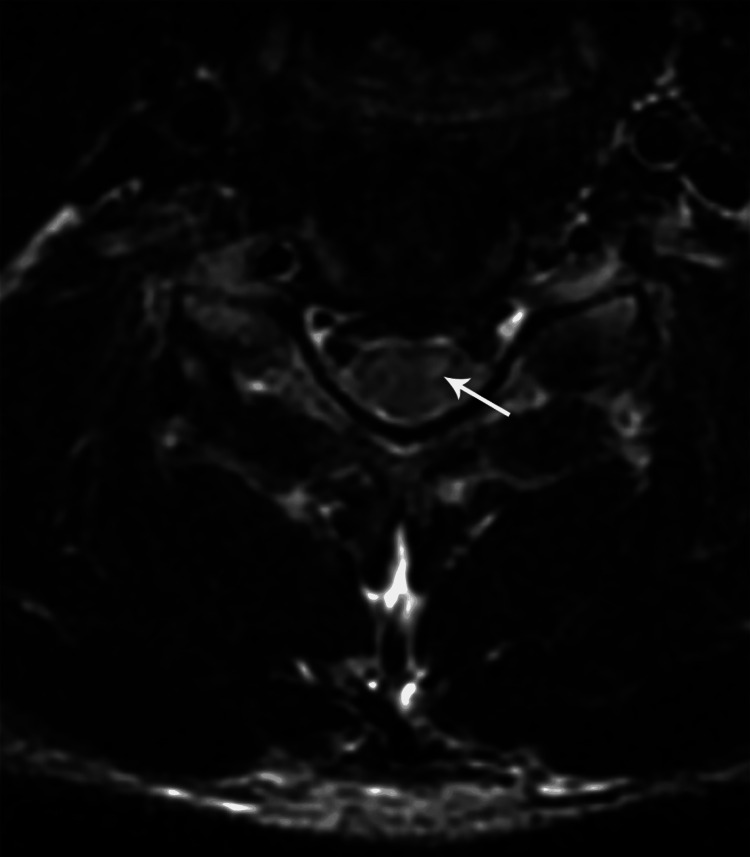
Axial T2W MR image of the cervical spine showing hyperintense intramedullary signal at the C5-6 level (case 1)

**Figure 13:**
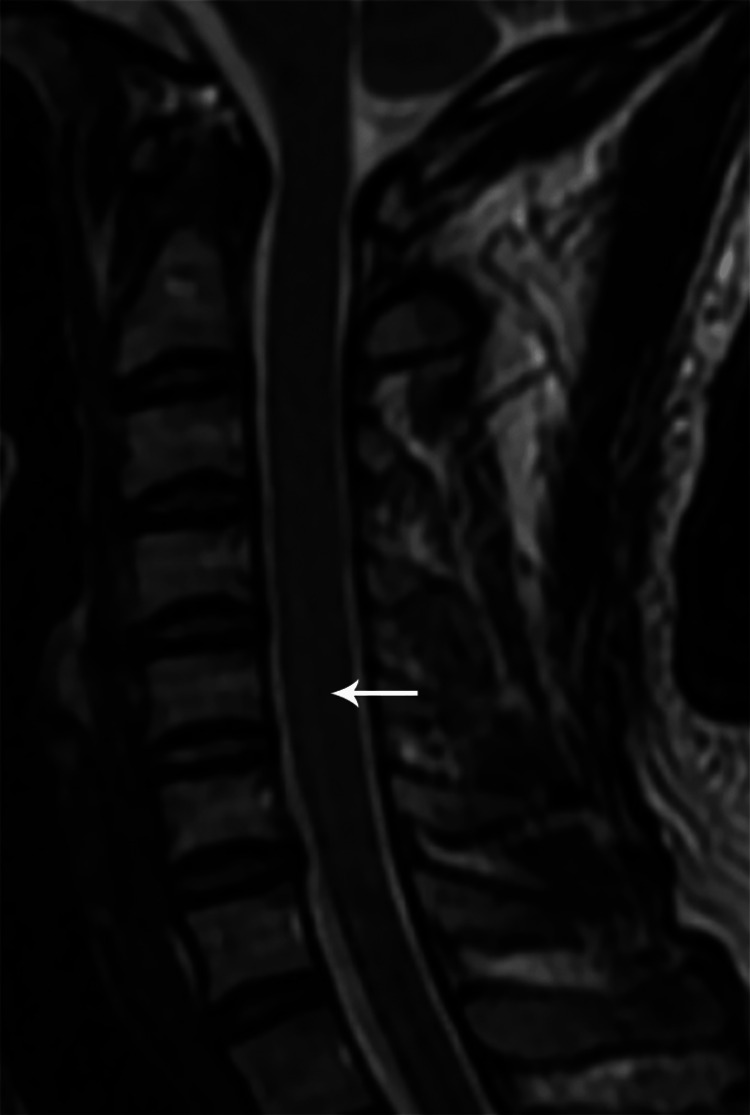
Sagittal T2W MR image of the cervical spine showing a short segment hyperintense intramedullary signal at C5-6 levels (case 3)

**Figure 14:**
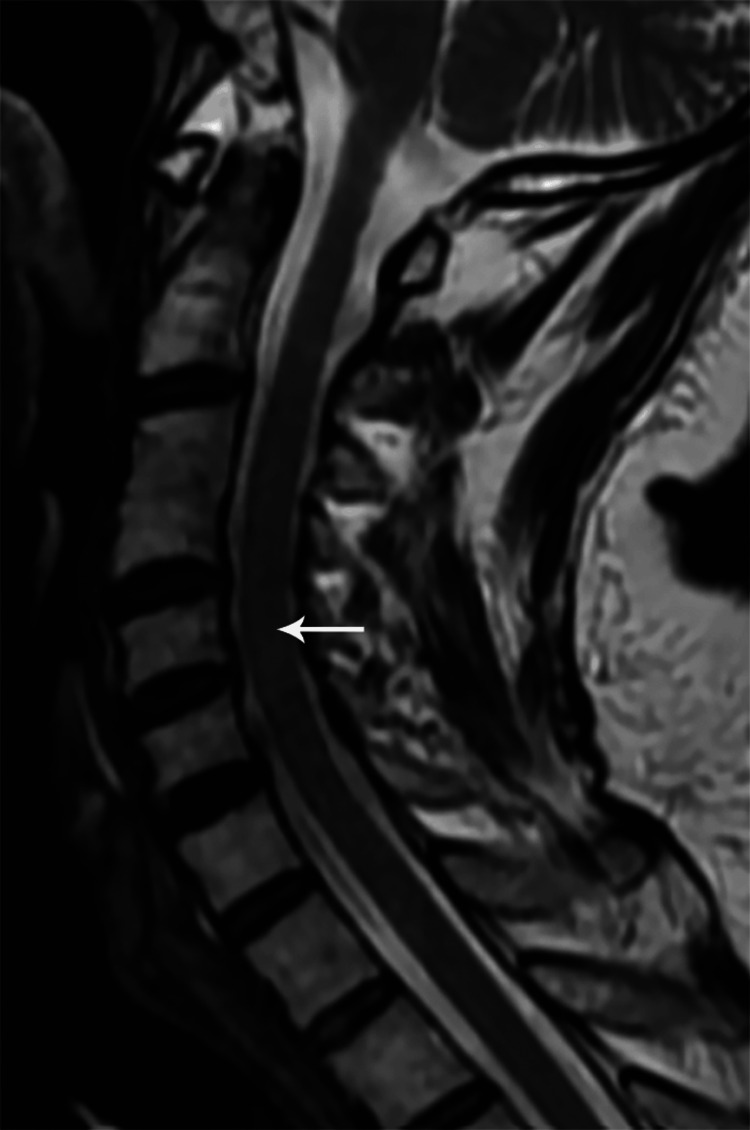
Sagittal T2W MR cervical spine image showing a long segment intramedullary hyperintense signal from C2 to C7 levels (case 5)

**Figure 15:**
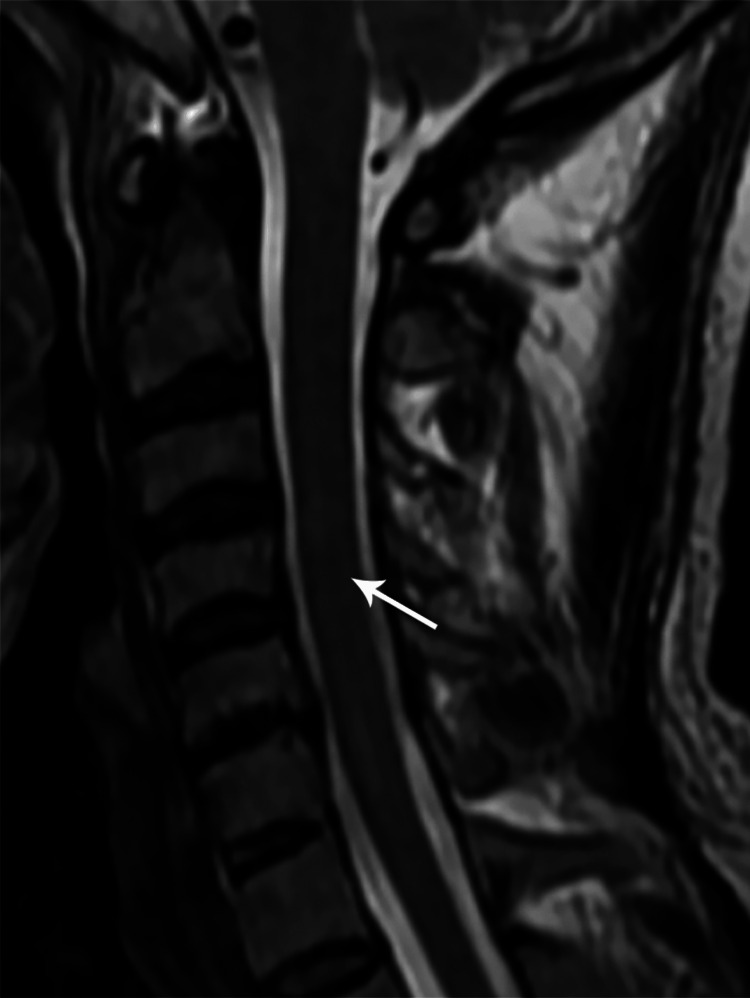
Sagittal T2W MR cervical spine image showing a long segment intramedullary hyperintense signal from C3 to C7 levels (case 6)

**Figure 16:**
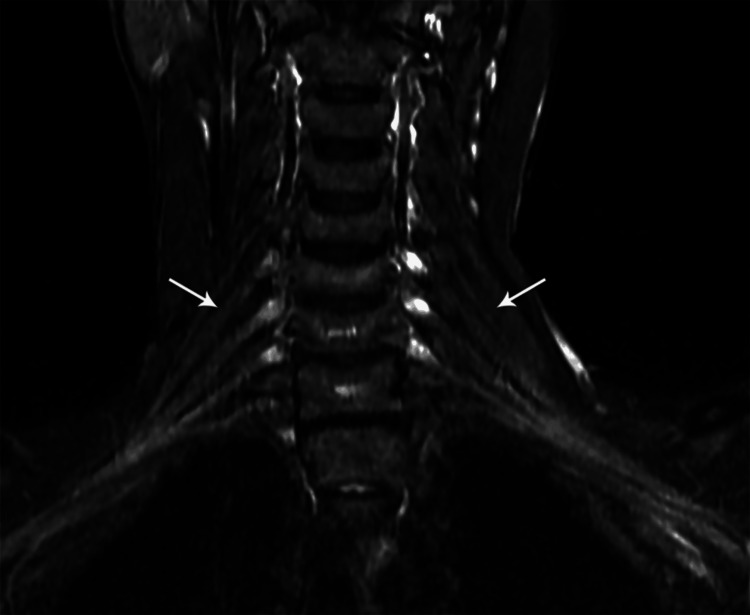
Coronal STIR MR image of the cervical spine showing thickened and hyperintense bilateral brachial plexus nerve roots C5 to T1 (case 2)

**Figure 17:**
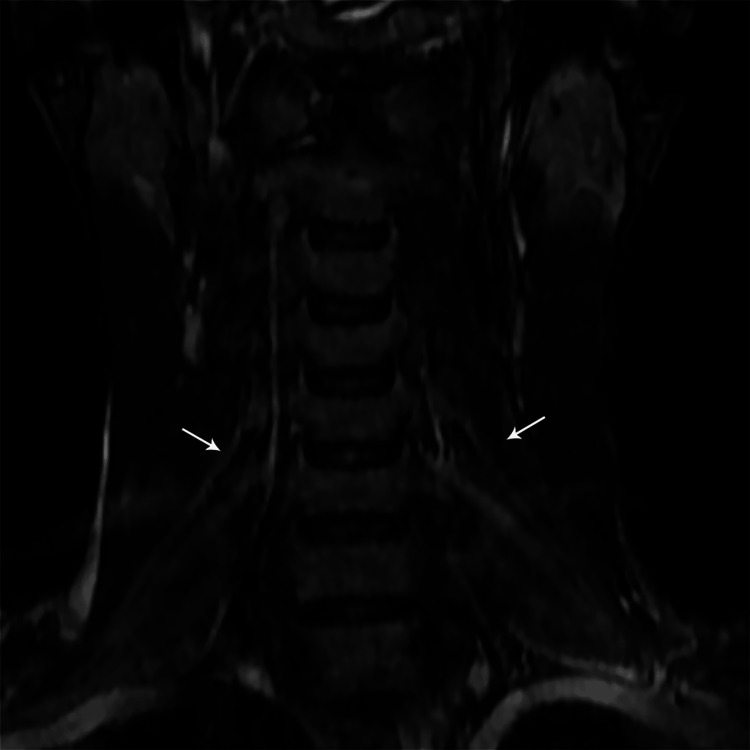
Coronal STIR MR image of the cervical spine showing thickened and hyperintense C5 & C6 brachial plexus nerve roots (case 3)

**Figure 18:**
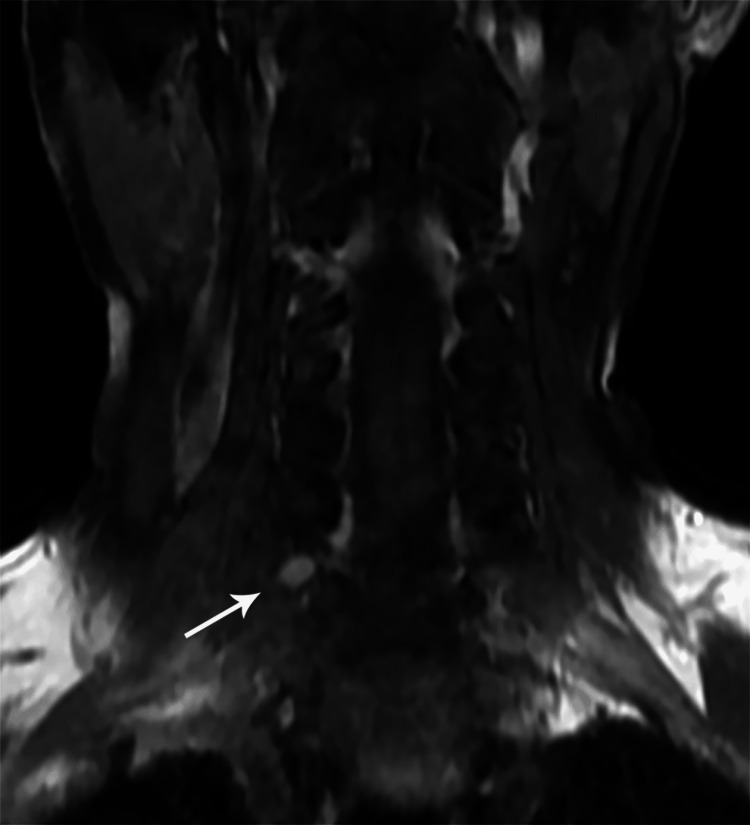
Coronal post-contrast T1W MR cervical spine image showing enlarged and hyperintense dorsal nerve root ganglion at C7 level on the right side (case 5)

**Figure 19:**
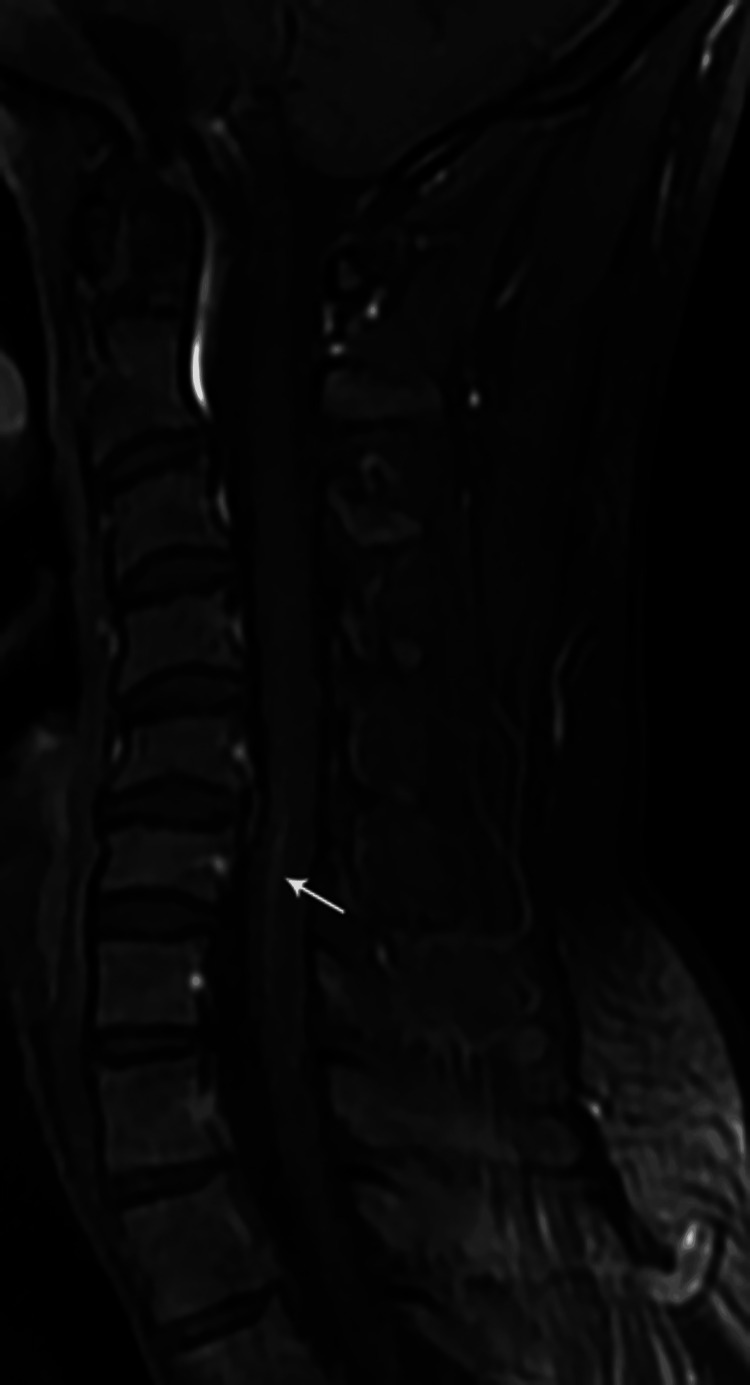
Sagittal post-contrast T1W MR image of the cervical spine showing contrast enhancement at the involved segments of the spinal cord at the C4-7 level (case 2)

Table 1 provides an overview of patients’ information.

**Table 1:** Summary of clinical, histological, and imaging characteristics of CNS leprosy patients CNS: Central nervous system, NCS: Nerve conduction studies, MRI: Magnetic resonance imaging, SIA: Signal intensity alteration, LSHS: Long segment hyperintense signals, MDT: Multi-drug therapy, BTHD: Borderline tuberculoid Hansen’s disease, MDT-MB: Multi-drug therapy-multibacillary, CP: Cerebellar peduncle, B/l: Bilateral, OIF: Oil immersion field, BLHD: Borderline lepromatous Hansen’s disease, BP: Brachial plexitis, DNRG: Dorsal nerve root ganglia, T1R: Type 1 reaction, BBHD: Mid-borderline Hansen’s disease, LLHD: Lepromatous Hansen’s disease, T2R: Type 2 reaction.

Age/ Gender	Cranial nerve involvement	Motor weakness/disability	NCS	Acid-fast bacilli in skin tissue (Wade-Fite stain)	Histological diagnosis Skin	MRI lesion(s) (SIA/LSHS)	Treatment provided	Follow-up MRI lesions one year post MDT
24y/M	None	Left ulnar claw	Left ulnar neuropathy	-	BTHD	C5- C6, Right middle cerebellar peduncle	MDT-MB with oral steroids	Minimal SIA C5-6 Persistent SIA in CP
26y/M	VII	B/l facial palsy, B/l claw hand and B/l claw toes	Sensory motor polyneuropathy	0-10 bacilli/OIF	BLHD	C4-C7 with bilateral BP(all roots) and bilateral ganglionitis (DNRG C8)	MDT- MB with oral steroids	Not Available
29y/M	VII	Left sided facial palsy	Sensory motor polyneuropathy	-	BTHD with T1 R	SIA C5-C6 with bilateral BP	MDT -MB with oral steroids	Persistent SIA C5 –C6 with Bilateral BP C5-6
33Y/M	VII	Left facial palsy, right hand and foot weakness	Mononeuritis multiplex	-	BBHD	Bilaterally symmetrical SIA at ponto-medullary junction at the level of facial nerve nuclei	MDT- MB with oral steroids	Complete resolution of SIA
40y/M	VII	Left facial palsy, left hand and left foot weakness	Non-contributory	>1000 bacilli/OIF	LLHD with T2R	C2-C7 with right sided ganglionitis (DNRG C6-C7)	MDT- MB with oral steroids	Reduction in SIA C3- C7 and right sided ganglionitis (DNRG C6)
44Y/M	VII	Right sided facial palsy	Sensory motor polyneuropathy	-	BTHD	C3 - C7 with bilateral ganglionitis (DNRG C5-7)	MDT- MBR with oral steroids	Residual short segment hyperintense signal C5-6 but persistent bilateral ganglionitis (DNRG C5-7)

After one year of completing MB-MDT, clinical examination revealed full recovery of Bell's phenomenon in three patients (Pt. No. 3, 5, and 6), while one patient (Pt. No. 4) showed partial improvement. One patient experienced a persistent but non-progressive ulnar claw (Pt. No. 1). Partial improvement in hand and foot weakness was observed in two patients (Pt. No. 4 and 5). Additionally, good control was achieved for T1R in one patient (Pt. No. 3) and T2R in another (Pt. No. 5).

**Figure 20:**
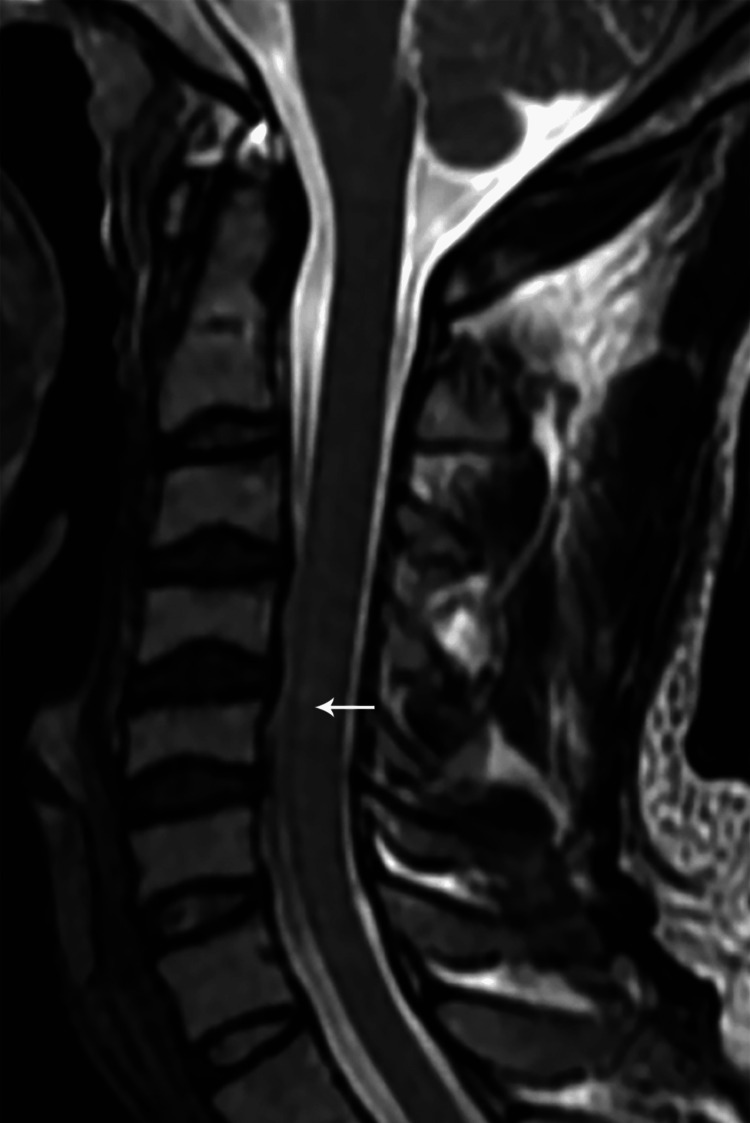
Sagittal T2W MR image of the cervical spine one year post-treatment showing residual short segment hyperintense intramedullary signal at the C5-6 level (case 1)

**Figure 21:**
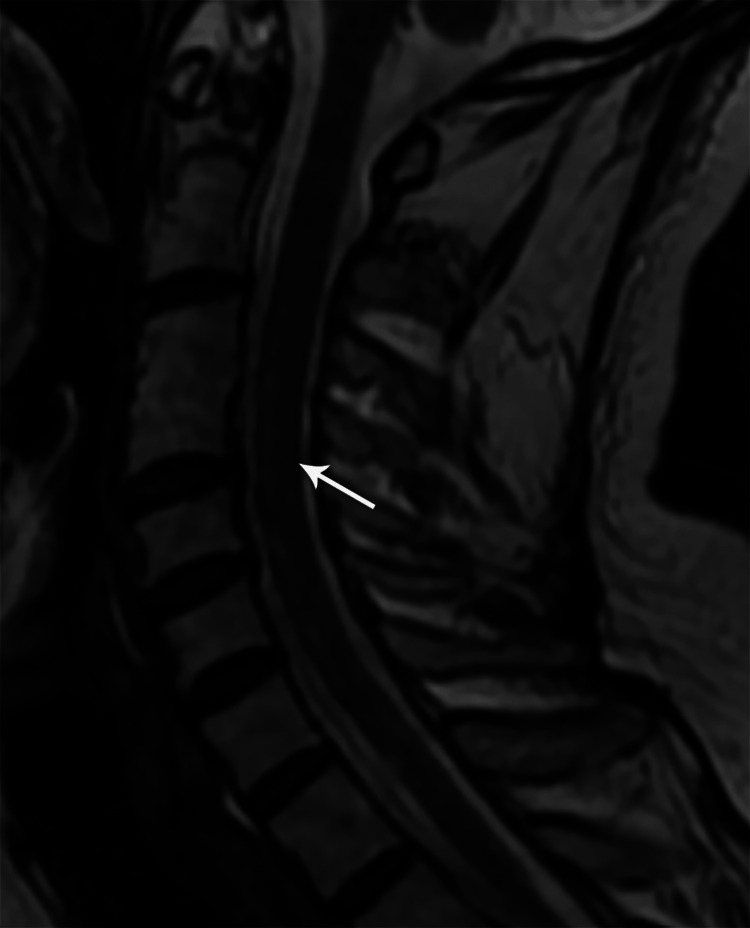
Sagittal T2W MR cervical spine image one year post-treatment showing residual long segment intramedullary hyperintense signal from C3 to C7 levels (case 5)

**Figure 22:**
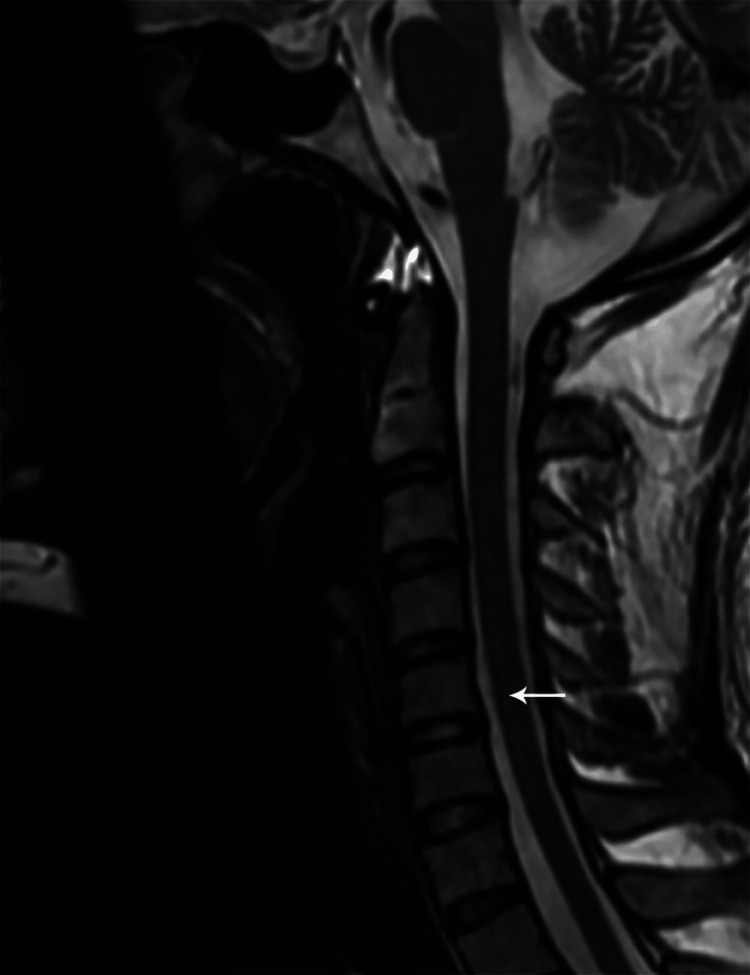
Sagittal T2W MR cervical spine image one year post-treatment showing a residual short segment intramedullary hyperintense signal at C5-C6 levels (case 6)

Follow-up MRI revealed persistent but decreased hyperintensity in the cervical cord in three patients (Figures 20-22).

**Figure 23:**
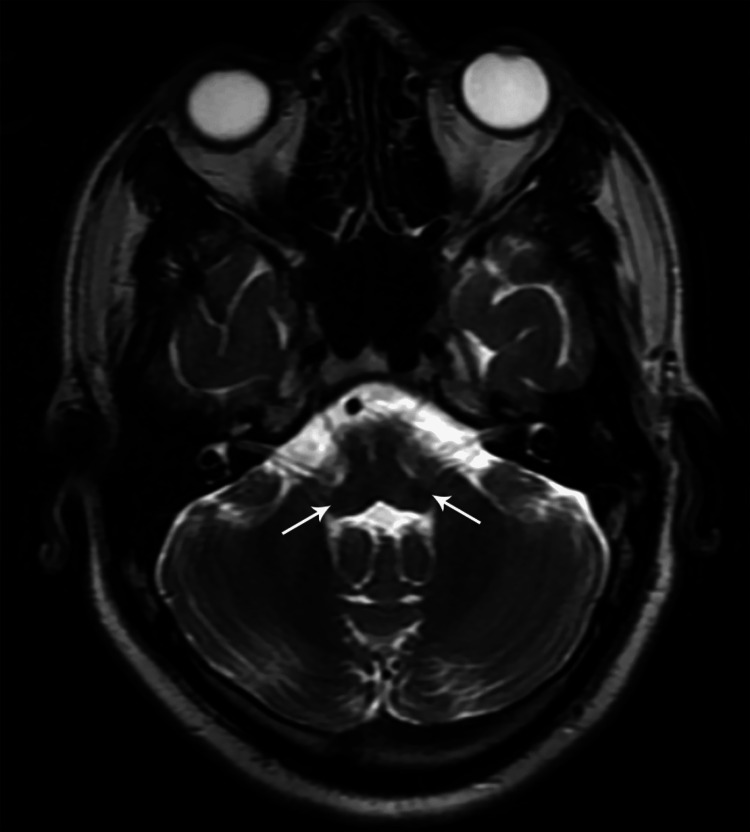
Axial T2W MR brain image one year post-treatment showing no residual hyperintense signal at the site of the lesion (case 4)

One patient demonstrated a reduced intensity of ganglionitis, while another showed sustained SIA in the middle cerebral peduncle. Additionally, one patient exhibited persistent bilateral ganglionitis. Notably, one patient had no observable changes in the cord and brachial plexus signals a year after treatment. Post one year of treatment, case 4 of BBHD showed complete resolution of brain lesions on MRI (Figure 23).

## Discussion

Brain MRIs in two patients showed high-intensity T2/FLAIR signals, with one case exhibiting lesions at the pontomedullary junction at the level of facial nerve nuclei and another in the right middle cerebellar peduncle. Polavarapu et al. [2] and Jabeen et al. [11] previously reported similar brain lesions linked to cranial nerve involvement [2,11]. Verma et al. documented lower motor neuron facial palsy with MRI showing bilateral symmetrical hyperintensities in the middle cerebellar peduncles in a single case [3]. Immunohistochemical studies confirm *M. leprae*'s presence in specific brain regions, suggesting a direct role in neuropathology [13]. Our study aligns with the work of Verma et al. [9] by presenting a case with brain imaging abnormalities despite the absence of cranial nerve deficits. This highlights the complex intricacies of leprosy presentation.

Cord lesions (C2-C7) were hyperintense on T2 imaging with patchy post-contrast enhancement, except for one case. Imaging changes in the dorsal root ganglion and brachial plexus were also noted, characterized by plexus thickening/ganglion enlargement with T2/short T1 inversion recovery (STIR) hyperintensity, supporting findings by Khadilkar et al. [14] and Rice et al. [15]. Jabeen et al. suggested that the thickening of the plexus may be due to inflammatory infiltrate, granuloma formation, and fibrosis, with hyperintensity potentially indicating myelin loss and an increased free water proportion [11]. It was seen, primarily in the borderline tuberculoid variant, aligning with our cases. In our study of six patients, encompassing two with lepra reactions, all exhibited brain and/or spinal cord lesions on MRI, regardless of lepra reaction type, which is in alignment with previous studies [10,16].

Various theories have attempted to explain these MRI abnormalities [2,17-20]. One theory, proposed by the Polavarapu group, suggests three potential pathways: direct retrograde infection, reactive changes in gray matter due to nearby nerve and root axonal transection, or an inflammatory immune response provoked by infection outside the central nervous system [2]. A few other studies supported the theory of extension of inflammatory changes from peripheral nerve trunks to the central nervous system, especially during a lepra reaction [21-23].

Post-treatment follow-up MRI revealed a partial reduction in cord signals with residual hyperintense signal and enhancement in three cases, while one case exhibited unchanged lesions. The resolution of brain lesions in a single case of BBHD at the level of the pontomedullary junction as a response to treatment further supports the notion of direct infection-related involvement of the cranial nerve nuclei. In another case, there was a partial resolution of cord signals along with dorsal nerve root ganglionitis. Cases involving brachial plexus demonstrated stable lesions on subsequent imaging.

A clinic-radiological dissociation was observed in this cohort of patients as MR signals persisted despite clinical improvement in some patients [2,11]. Further investigation is needed to determine whether a cause-effect relationship exists between these findings. The persistence of signals without reduction in some patients might suggest permanent gliosis [24] within specific subgroups, warranting further exploration in future research endeavors.

A recent systematic review by Garg et al. concluded that CNS is affected in cases of leprosy, even without cutaneous manifestations, covering lepra reactions associated with different types of leprosy across the spectrum [10].

CNS involvement in leprosy is not observed in all patients but only in a few. This prompts researchers to delve deeper into the genetic aspects of both patients and bacteria to understand the risk factors associated with CNS involvement.

A small sample size remained the limitation of our study. 

## Conclusions

This study revealed diverse radiological findings in leprosy patients who underwent dedicated neuroimaging. A craniospinal MRI may reveal signal intensity alterations in leprosy patients, especially in those who have lower motor neuron-type facial palsy. However, the significance of these findings needs to be validated through future well-designed prospective studies.
